# Multiple sclerosis epidemiological trends in Italy highlight the environmental risk factors

**DOI:** 10.1007/s00415-021-10782-5

**Published:** 2021-09-27

**Authors:** M. Puthenparampil, P. Perini, R. Bergamaschi, M. Capobianco, M. Filippi, P. Gallo

**Affiliations:** 1 Department of Neurosciences, University of Padua, via Giustiniani 5, 35128 Padua, Italy; 2 Multiple Sclerosis Centre, Azienda Ospedaliera di Padova, Padua, Italy; 3 Multiple Sclerosis Research Centre, IRCCS Mondino Foundation, Pavia, Italy; 4 Centro di Riferimento Regionale Sclerosi Multipla (CReSM), SCDO Neurologia, AOU S. Luigi Gonzaga, Orbassano, Turin, Italy; 5 Unit of Neurology, Unit of Neurorehabilitation and Neurophysiology Service, IRCCS San Raffaele Institute, Milan, Italy; 6 Multiple Sclerosis Centre, University Hospital of Padua, via Giustiniani 5, 35128 Padua, Italy

**Keywords:** Multiple sclerosis, Epidemiology, Incidence, Prevalence

## Abstract

Italy is definitely a high-risk country for multiple sclerosis (MS). Over the last 50 years, several epidemiological studies, including longitudinal surveys, have disclosed that MS incidence and prevalence in Italy mainland and Islands (Sardinia and Sicily) have progressively increased, picturing a semi-parabolic curve. Based on the comprehensive scrutiny of 58 papers, we conclude that the latitude risk gradient does not fit to the Italian map of MS. The genetic heterogeneity of the Italian ethnicities, that likely forms the basis of MS predisposition, does not account for the dramatic increase of MS incidence and prevalence observed in Italy over the last half century that, rather, seems better explained by the effect of environmental factors.

**Supplementary Information:**

The online version contains supplementary material available at 10.1007/s00415-021-10782-5.

## Introduction

The existence of a latitude-related risk gradient is accepted as a milestone of multiple sclerosis (MS) scientific culture [1] and interpreted, depending essentially on the author’s personal point of view, either as the effect of the genetic background or the consequence of environmental factors. However, epidemiological observations in several European Countries do not fit to the latitude theory but rather suggest that the variability in MS risk is a more complex phenomenon, characterized by significant, often unexpected, intra-regional variations. Indeed, the global dispersion of MS prevalence (ranging from 5.2 to 335) and incidence (ranging from 0.5 to 20) is largely independent of the latitude [2]. Nevertheless, despite repeated indications to re-considering the latitude effect [3, 4], the MS Atlas still represents the uneven world distribution of MS as a latitude-related phenomenon (www.msif.org).

This review addresses two major questions rising from five decades of epidemiological studies conducted in Italy’s mainland, Sicily and Sardinia, namely: (1) is the latitude hypothesis still convincing or should be re-considered or even definitely abandoned? (2) do the observed epidemiological trends suggest that environmental factors play a primary role in increasing MS risk, thus moving to the background the population genetics?

## Methods

Cochrane Library, Web of Science, and PubMed databases were used for systematic literature retrieval by matching and intersecting the following keywords: multiple sclerosis, epidemiology, incidence, prevalence, Italy, Sardinia, Sicily. After removing duplicates, the titles and abstracts were screened independently by two investigators to evaluate whether they were eligible or potentially eligible literature or not. Only full-text papers were considered and meeting or conference abstracts were excluded. All the available literature, up to November 30, 2020, was screened by two independent investigators (MP and PG). If there were any differences between two investigators’ screening, differences would then be discussed together with a third investigator of the panel to meet an agreement. Papers were excluded if they met one of the followings: (i) hypothesis, case reports, review articles, letter to editor comments, case reports; (ii) studies available only in abstract form; (iii) unclear methodology; (iv) unclear statistics. After scrupulous selection, we collected and analyzed the data of 58 papers that were considered relevant for this analysis. Twenty-eight provided data from 35 prevalence or incidence time-points for Italy’s mainland, 13 for Sardinia and 17 for Sicily.

## Results

### MS in Italy’s mainland

**Fig. 1:**
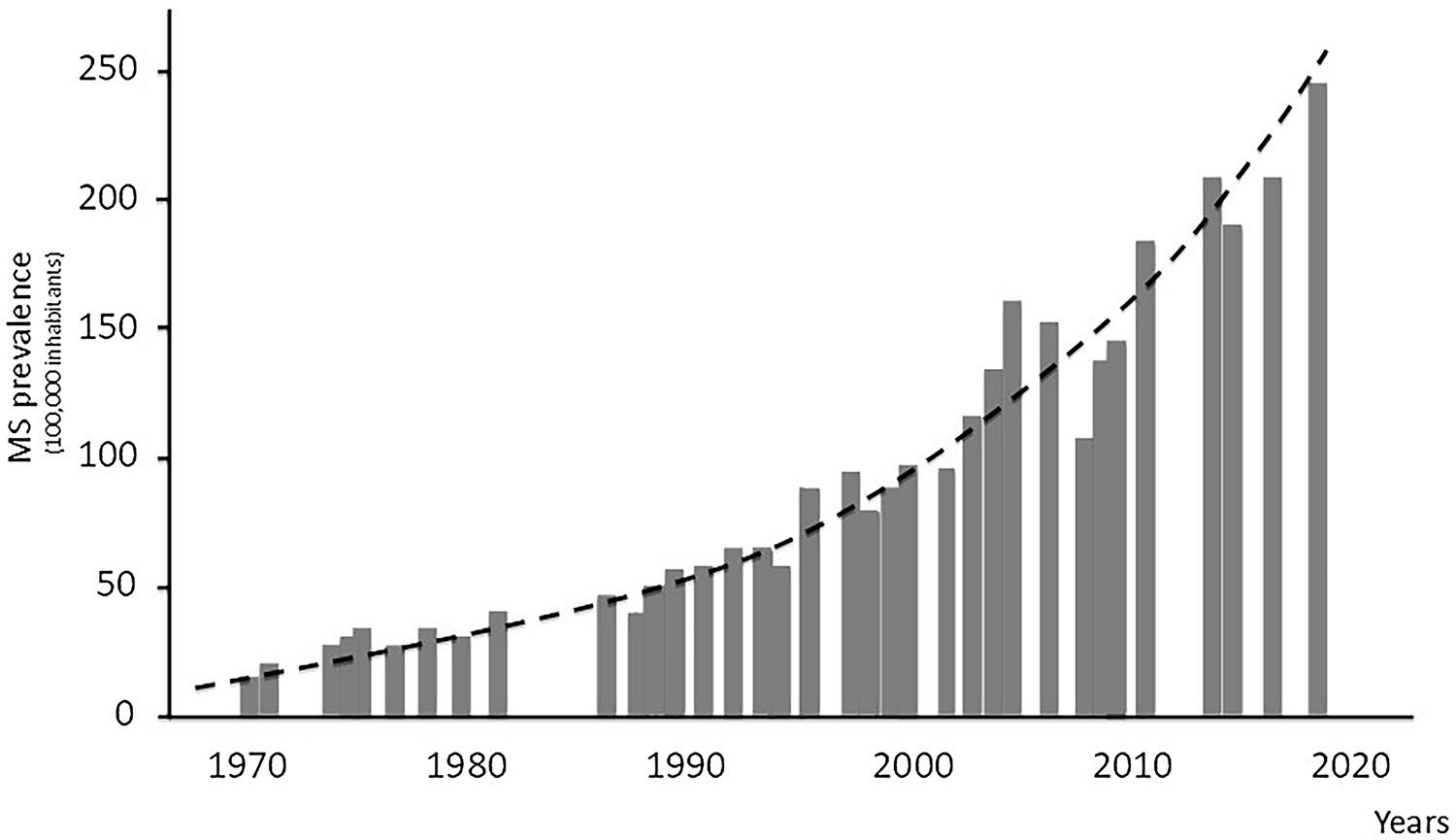
Trend of MS prevalence in Italy over the last 5 decades. Bars report MS prevalence values described in the epidemiological studies conducted in Italy mainland over the last 50 years (Ref. 8–10, 12–23, Supplementary Bibliography, 1–10). The increase in MS prevalence describes a semi-parabolic curve, without plateau or slowing-down periods during the last 5 decades

The data on MS prevalence collected in Italy’s mainland over the last half century describe a semi-parabolic curve, with no plateau or slowing-down period even when these were reasonably expected (Fig. 1). Indeed, in the first part of the ‘80ies, the majority of the Italian MS Centres established the modern MS diagnostic workup thanks to the availability of magnetic resonance imaging (MRI), advanced cerebrospinal fluid (CSF) examination, visual evoked potentials, more extensive immunological evaluation and the application of the Poser’s diagnostic criteria [5]. It would have expected a marked reduction of the diagnostic gap in the following years, with an initial increase in incidence and prevalence, followed by a plateau period with a stabilization of the epidemiological figures. This did not happen. Even after the settlement of the McDonald’s criteria [6] and the subsequent modifications up to the 2017 revised criteria [7], no change in the epidemiological trend was observed, thus indicating that the increase in MS risk is probably true and not the mere effect of the improvements in the diagnostic abilities.

**Fig. 2:**
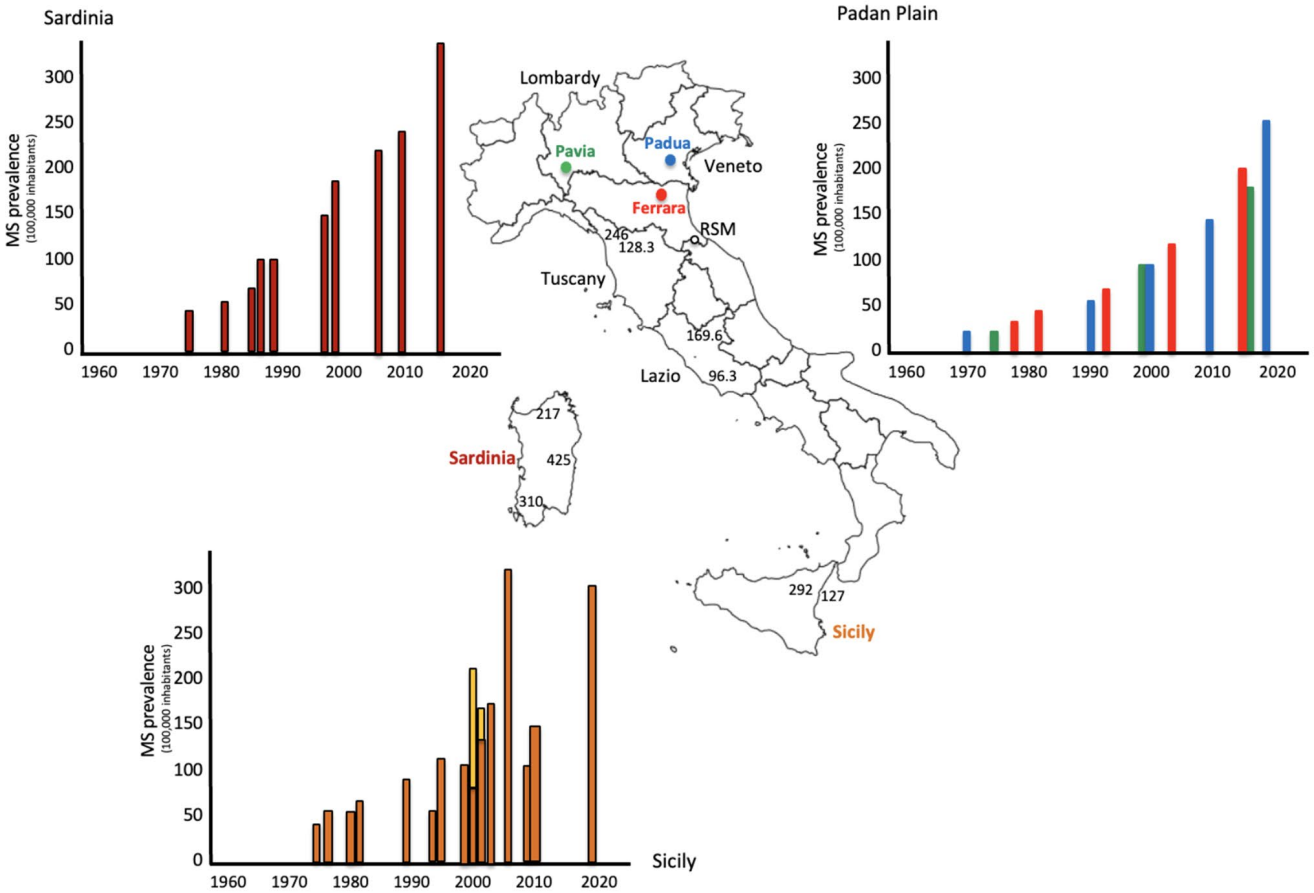
Trends of MS prevalence in Padan Plain, Sardinia e Sicily. Graphics report the prevalence trend observed in the Padan Plain (Ref. 10, 12–18), Sicily (Ref. 35–44, Supplementary Bibliography, 11–15), and Sardinia (Ref. 24–31, Supplementary Bibliography, 16–18) over the last 50 years. In the map of Italy, loco-regional differences in MS prevalence are reported for Tuscany, Lazio, Sicily and Sardinia. *RSM* Republic of San Marino. See text for details

Nevertheless, significant differences in MS prevalence are noticed not only between Regions, but also within the same Region and even within the same Province. In 2015, the estimate number of MS patients in Italy was 109,000 [8], with a mean prevalence value of 182/100,000, but ranging from 96.3/100,000 in the City of Latina in Lazio (a Region in the centre of the peninsula) [9], to 180 in the Province of Padua [10], and to 425/100,000 in the district of Nuoro/Ogliastra in Sardinia [11]. Worthy of particular interest are the data rising from the longitudinal surveys conducted in the Padan Plain (i.e., the Po Valley), one of the most polluted areas of Europe. The prevalence of MS in Ferrara, a City located in the South-east part of the Plain (Fig. 2), progressively increased from 26.9/100,000 in 1978 to 46.1/100,000 in 1981, 69.4/100,000 in 1993, 120.9/100,000 in 2004 and 194.9/100,000 in December 31, 2016 [12, 13]. This trend was strikingly superimposable to that observed in the Province of Padua, located about 70 km North from Ferrara, where MS prevalence progressively increased from 16/100,000 in 1974 to 45.7/100,000 in 1990, 80.5/100,000 in 1999, 139.5/100,000 in 2009, 180/100,000 in 2015 and 220/100,000 in 2020, with a peak of 245/100,000 in urban area of Padua [10, 14–17]. In addition, three epidemiological surveys conducted in the Province of Pavia, a district of Lombardy located in the centre of the Padan Plain, at the same latitude of Padua and Ferrara, gave prevalence values also strictly superimposable to those observed in Ferrara and Padova, namely 16/100,000 in 1976, 86/100,000 in 2000 and 169.4/100,000 in 2016 [18] (Fig. 2).

Worth of interest are the results of the epidemiological studies done in the Republic of San Marino, a little independent State in the Northern Italy, 150 km South from Ferrara, whose population (32,876 inhabitants) is almost exclusively Caucasian (composed by natives in San Marino, 85%, and Italy, 10%). In this State, MS prevalence increased with the same trend: 51.6/100,000 in 1984 [19], 121/100,000 in 2005 and 203/100,000 on December 31, 2014 [20].

Intriguingly, significant variations in MS incidence and prevalence were observed in neighbouring areas within the same Regions. In Tuscany, where the mean MS prevalence was settled at 208.7/100,000 on January 1, 2017 [21], a striking local difference was observed between the neighbouring local health care agencies (LHAs) of Viareggio (128.3/100,000) and Massa and Carrara (246/100,000), despite these two LHAs are located in front of the Tyrrhenian Sea and share ethnicity and economy. Very similar loco-regional differences were also observed in Lazio, a Region confining with Tuscany, where the mean prevalence of MS on December, 31st 2011 was 130.5/100,000, ranging from 96.3 in the City of Latina (a coastal city of 126,00 inhabitants) to 169.6 in the City of Rieti (a hilly city of 47,000 inhabitants) [22]. In the Padan Plain, where pollution constitutes a dramatic health care issue, significant differences in MS prevalence have been recently observed between the urban areas and the neighbouring hilly areas, both in the Province of Pavia and in the Province of Padua, and associated with the exposure to particulate matter (PM) 2.5 [10, 18, 23].

A number of incidence studies have also been performed in Italy mainland. However, longitudinal data are available only from a few studies. Methodologically, it has to be stressed that incidence studies based on small population cohorts and/or on short periods of observation (i.e., < 5 years) generally produce overestimated figures. These studies were not considered in this review. In other studies, the diagnostic criteria and/or the sources of cases were not adequately described. In general, the most accurate incidence rates available come from prospective studies based on the direct screening of the patients. Retrospective case-finding procedures, that include at least 2 years of post-observational period in order to cover the actual diagnostic gap, provide more useful incidence estimations. Even taking into consideration the limitations above described, a progressive increase of MS incidence occurred in Italy mainland as well as in the islands over the last half century. Indeed, since the ‘60ies to the ‘90ies very similar increase in MS incidence were scored in the Province of Padua (937,000 inhabitants) where MS incidence increased from 0.9/100,000/year to 5.5/100,000 [16], in the Province of Ferrara (351,400 inhabitants), from 2.3/100,000 in the ‘80 to 4.09/100,000 in the '90ies, and in the Province of Sassari in Sardinia (334,000 inhabitants), from 1.1 to 5.8/100,000 [24]. Since incidence data are expressed as mean values of a 5- or 10 year periods, their plotting in a graph is unworkable. However, the increase in incidence also describes a semi-parabolic curve, with no slow-down period.

### Sardinia

Several studies [3, 25–31] performed between 1996 and 2011 have definitely estimated the prevalence of MS in Sardinia to be much higher compared to that of other Regions of Italy mainland. Since the early ‘70ies, MS prevalence in Sardinia has constantly increased reaching the mean value of 330/100,000 [11], that makes this Island one of the European geographic areas with the highest MS risk, together with Shetland and Orkney Islands [32].

However, MS prevalence throughout the island is far to be homogeneous with values ranging from 217 in the district of Olbia-Tempio to 425 in the districts of Ogliastra and Nuoro, located less than 100 km South [11] (Fig. 2). Sardinia has remained a quite isolated island for millennia and the genetic background of the Sardinian people differs from that of the Italian population of the mainland [33], a factor that likely accounts for the higher risk of MS (as well as of other autoimmune diseases) observed in the island since the earliest epidemiological surveys carried in the ‘60ies and ‘70ies. However, the striking intraregional differences and the recent dramatic trends do not find a genetic explanation. Indeed, over the last decade the mean MS prevalence in Sardinia raised from 224/100,000 in 2009 to 330/100,000 in 2016 [11]. This increase is very similar to that observed in the longitudinal surveys carried out in the Padan Plain during the same interval of time (Fig. 2).

### Sicily

As for Sardinia, the surveys conducted in different Sicilian localities have disclosed a high-risk for MS in this island and pointed out that the latitude hypothesis does not fit to the epidemiological trends of the disease in this island (Fig. 2). Although the cities of Monreale and Enna, characterized by high epidemiological values [34–37], had a prolonged Norman domination, suggesting a genetic background coming from Northern Europe, high MS prevalence was observed in areas having different geoclimatic features [38]. Moreover, local intra-regional differences suggesting a primary role of environmental factors have been reported. Of interest is the significant difference in MS risk in the City of Catania as compared to the very near (50 km far) small town of Linguaglossa. Indeed, from 1975 to 2004 in the city of Catania, MS incidence increased from a mean of 1.3/100,000 during the quinquennium 1975–1979 to 7.0/100,000 during 2000–2004, while in Linguaglossa (5200 inhabitants) the average annual onset-adjusted incidence risk was 19.4/100,000 during the 15 year period ranging 1991–2006 [39–42]. In the same geographic area, worthy of consideration are the epidemiological figures observed in the City of Biancavilla (23,700 inhabitants), about 30 km North-west from Catania, where MS incidence increased from 4.5/100,000 in the period 1992–1996 to 16.8/100,000 in the period 2012–2018. These high incidence rates, however, should be taken with caution given the very low denominator and the short intervals considered. The most recent estimates of MS prevalence gave values of 292.3/100.000 in Biancavilla and 127.1/100,000 in Catania [43]. A more recent geo-epidemiological study on MS incidence in the Mt Etna region has confirmed significant loco-regional differences and further stressed the role played by environmental factor probably related to volcano-derived heavy metal and gases.

## Discussion

MS is an autoimmune disease, i.e., the results of a complex interaction of genetic and environmental factors. A common polygenic background is supposed to predispose to autoimmunity [44], but its origin and how its heterogeneity is shaped by environmental factor remain not clarified. A recent study on the genomic history of the Italian population, based on high-coverage whole-genome sequences, has pointed out the genetic diversity of Italians, with peculiarities characterizing the populations living in the North and in the South of Italy [45]. The genetic heterogeneity of Italian populations/ethnic groups likely results from multiple ancient migrations along the Italian peninsula and adaptive events (i.e., climate- and environmental-related selective pressures) that started to accumulate after the Late Glacial Maximum, which ended approximately 19,000 years ago. This genetic heterogeneity forms the basis to understand the deep causes that predispose Italians to a number of diseases [45] as well as the differences in MS susceptibility observed between populations living in well-defined geographic areas, e.g., Padana Plain versus Sardinia versus Sicily. However, it does not explain the epidemiological trends observed over the last 5 decades all over the Country, characterized by a dramatic increase of MS risk in almost all the Regions, but with striking local differences between neighbouring Regions and even within the same Region. Neither the latitude gradient nor the genetic background can be considered plausible explanations for these findings.

On the other hand, many environmental factors have been suggested to play a role in either triggering MS or modulating the subsequent disease course, but results vary substantially between studies [46]. In addition, no demonstration that one or more specific risk factors have been successfully targeted or modified, thus reducing the disease risk and course, has been produced. Among the modifiable risk factors, EBV virus infection, smoking, vitamin D and sun exposure, obesity and diet, occupational-related toxins and pollutants are worthy of particular consideration on the base of the consistency of the available literature [47] and the possibility of planning strategies aimed at reducing their impact on the diseases.

The emerging role of air pollutants in the Padan Plain, that may partly explain the MS epidemiological trends described above, is worth of consideration. Indeed, increasing evidence strongly associates PM to MS risk in this geographic area that is one of the most polluted Region of Europe. Studies independently conducted in the Provinces of Padua and Pavia showed quite concordant results and indicated a strong association between MS and PM. In Lombardy Region, a fourfold PM_10_ concentration associated with higher risk of MS relapses and brain magnetic resonance imaging (MRI) inflammatory activity [48]. In the Province of Padua, identified by the European Environmental agency as the most polluted city of Europe for many years, MS prevalence was found to be associated with PM_2.5_ exposure [10, 23]. More recently, significant variations in MS prevalence between urban areas and adjacent hilly areas associated to PM_2.5_ concentrations in both Provinces [10, 18]. In addition, a recent study in Piedemont region (North-West of Padan Plain) further disclosed a relationship between MS prevalence and levels of urbanization [49]. Thus, air quality may be identified as one of the possible risk factors for MS in the Padan Plain.

An increasing amount of data supporting a role for PM in MS risk has risen from studies conducted in other Countries, at different latitude, and recently object of a meta-analysis [50]. A pivotal study conducted in southern Finland found that air concentrations of PM_10_, CO_2_, NO and SO_2_ were associated with MS prevalence and the risk of relapses [51]. A study in Tehran [52] found a significant correlation between clustering of MS and patterns of PM_10_, SO_2_, and NO2 concentrations. An association between PM_10_ exposure and risk of MS relapses was observed in Strasbourg (France) [53]. Particularly worth of interest is a recent US study, reporting that PM_2.5_, CO, SO_2_, and Pb values in the upper quartiles of emissions were all significantly related to higher odds for pediatric-onset MS [54].

Furthermore, a recent study has investigated the risk of MS in the industrialized area of Southwest Finland, having the city of Turku as Capital, compared to that the green region of North Karelia, located at the same latitude. A significant higher incidence (12.1 vs 6/100.000 year) and prevalence (275 vs 167/100.000 at the end of 2016) of MS was observed in the more polluted region of Southwest Finland [55]. In Turkey, a significant difference in MS prevalence was recently observed between Karabuk, a highly industrialized and polluted city (107.1/100,000) and Akcakoca, a touristic coastal city located at the same latitude, 200 km East on the Black Sea (41.5/100.000), strongly suggesting that pollution could be a primary environmental factor determining the marked difference in MS risk, since no other significant differences could be found between the two examined populations (i.e., genetics, vitamin D levels, sun exposure, economical level, etc.) [56].

Regional differences in MS prevalence were also observed in Croatia in the ‘90ies, ranging from 194/100,000 in Gorski Kotar to 27/100,000 in the neighbour Istria [57]. In this Country, confining with North-east Italy, MS prevalence raised up to 143.8/100,00 in 2015, i.e., more than twice compared to the value (60/100,000) estimated in 2008 [58].

**Fig. 3:**
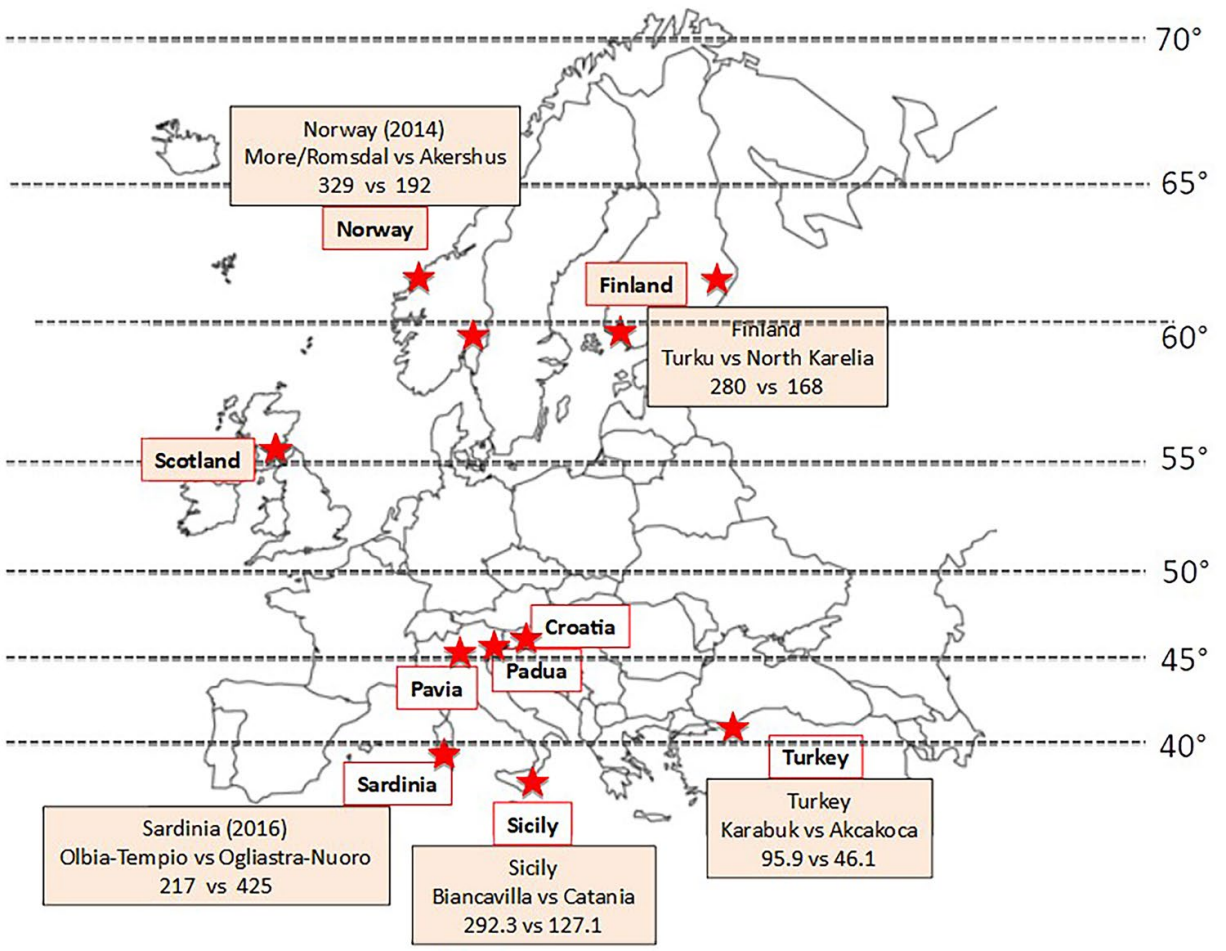
Locoregional differences in MS prevalence or incidence in Europe. Regional differences in MS prevalence and incidence that question the latitude gradient are observed throughout Europe. See text for details

As a matter of fact, striking loco-regional differences in MS prevalence are observed in several European Country, both in the Mediterranean area and in the Scandinavian area (Fig. 3).

Nevertheless, the idea that a single environmental factor may play a dominant role in MS in all the geographic areas and ethnic groups is questioned by the regional variation in the incidence rate observed in other European regions. For instance, in Scotland in the period 2010–2017 [59] MS incidence rate was almost double in Tayside county (12.81) than in the very close Lothian county (6.85), both located at 57° latitude (Fig. 3). Worth of interest is the finding that the urban areas of Great Glasgow and Edinburgh had the lowest incidence values, suggesting that other factors, rather than PM, likely play a major role in these Cities. Indeed, the comparison of MS prevalence between urban and rural areas in the district of Telemark, Norway, has recently disclosed higher values in rural areas (316.2 vs 250.4), further questioning the latitude gradient as well as the role of sunlight and diet [60]. While questioning the latitude-related effect, all these observations further point out the concept that environmental risk factors form puzzles that vary from Country to Country, region to region.

## Conclusions

Incidence and prevalence of MS in Italy mainland and Islands (Sicily and Sardinia) have constantly increased all over the Country over the last 5 decades, with a clear latitude-independent trend. In Italy, the latitude-effect is also questioned by the significant differences observed between areas located at the same latitude or even adjacent, a finding shared with others European Regions.

Although the genetic background may account for the basic predisposition to develop MS, that characterizes the different Italian populations, the epidemiological data collected in Italy over the last half century indicate that environmental factors play a major role in determining the risk of developing MS in the Caucasian people living in Italy.

## Supplementary Information

Below is the link to the electronic supplementary material.

Supplementary file1 (DOCX 15 KB)
