# Supplementary material for: Multiple sclerosis epidemiological trends in Italy highlight the environmental risk factors
Source: J Neurol. 2021 Sep 27;269(4):1817–24. doi: 10.1007/s00415-021-10782-5 (PMC8940874; doi:10.1007/s00415-021-10782-5)
Supplement: Supplementary file 1 — Supplementary file1 (DOCX 15 KB) [file 415_2021_10782_MOESM1_ESM.docx]

**Supplementary Bibliography**

1. Bellantonio P, Iuliano G, Di Blasio F, Ruggieri S: Prevalence and incidence of multiple sclerosis in Campobasso (Molise region chieftown,southern Italy). Clin Neur Neurosurg 2013; 115: 1806–1808.
2. Govoni V, Casetta I, Granieri E, Veronesi V,Malagù S, Tola MR, Paolino E, Fainardi E, Monetti VC, Aiello I: Mortality study on multiplesclerosis in the province of Ferrara, northern Italy, 1968 through 1989. ActaNeurol(Napoli) 1993; 15: 161–176.
3. Granieri E, Malagù L S, Casetta I et al. Multiple sclerosisin Italy. A reappraisal of incidence and prevalence inFerrara.Archives of Neurology 1996; 53: 793–798.
4. Maddestra M, Sabbatini S, Paci F, Paci A. Epidemiological survey of multiple sclerosis in the province of Terni. Neuroepidemiology 1998; 17: 54.
5. Malatesta G, Gabriele A, Macor S et al. Diffusion of multiple sclerosis in two Italian provinces, Chieti-Pescara. Italian Journal of Neurological Sciences 1991; 12(Suppl. 5): 83.
6. Meucci G, Bianchi F, Rossi G. Indagine sulla prevalenza della sclerosi multipla nella USL n.17 del Valdarno inferiore. In: 7th Italian Congress of Neuroepidemiology. Tipografia Umbra Perugia, 1992; p. 35.
7. Sironi L, Mamoli A, D’Alessandro G, Camerlingo M, Bottacchi E: Frequencyof multiple sclerosis in Valle d’Aosta, 1971–1985. Neuroepidemiology1991; 10: 66–69.
8. Sironi L, D’Alessandro G, Corso G, BottacchiE.Epidemiologyof multiple sclerosis in Valle d’Aosta, Italy.Multiple Sclerosis 1997; 3: 283.
9. Totaro R, Marini C, Cialfi A et al. Prevalence of multiple sclerosis in the L’Aquila district, central Italy. Journal of Neurology, Neurosurgery and Psychiatry 2000; 68: 349–352.
10. Iuliano G. Prevalence of multiple sclerosis in Salerno Italy: preliminary data in a study on general practitioners. Italian Journal of Neurological Sciences 1999; 20(Suppl. 4): S186.
11. Dean G, Grimaldi G, Kelly R, Karhausen L. [Multiple sclerosis in southern Europe. I: Prevalence in Sicily in 1975.](https://pubmed.ncbi.nlm.nih.gov/490087/?from_term=multiple+sclerosis+prevalence+sicily&from_sort=date&from_page=5&from_pos=8) J Epidemiol Community Health. 1979 Jun;33(2):107-10.
12. Dean G, Savettieri G, Giordano D, Butera C, Taibi G, Morreale S, Karhausen L. [The prevalence of multiple sclerosis in Sicily. II: Agrigento city.](https://pubmed.ncbi.nlm.nih.gov/7299336/?from_term=multiple+sclerosis+prevalence+sicily&from_sort=date&from_page=5&from_pos=3) J Epidemiol Community Health. 1981 Jun;35(2):118-22.
13. Grasso AA, Reggio A, Marano P, Florio S, Scordino N, Cappello S, Nicoletti F. [Epidemiological survey of multiple sclerosis in Catania city.](https://pubmed.ncbi.nlm.nih.gov/1601629/?from_term=multiple+sclerosis+prevalence+sicily&from_sort=date&from_page=4&from_pos=7) Ital J Neurol Sci. 1992 May;13(4):301-9. Neurology. 2001 Nov 27;57(10):1891-3.
14. Nicoletti A, Messina S, Bruno E, Mostile G, Quattrocchi G, Raciti L, Dibilio V, Cappellani R, D'Amico E, Sciacca G, Lo Fermo S, Paradisi V, Patti F, Zappia M.[Risk factors in multiple sclerosis: a population-based case-control study in Sicily. Background and methods.](https://pubmed.ncbi.nlm.nih.gov/27488302/?from_term=multiple+sclerosis+prevalence+sicily&from_sort=date&from_pos=4) Neurol Sci. 2016 Dec;37(12):1931-1937.
15. Salemi G, Ragonese P, Aridon P, Scola G, Saporito V, Conte S, Savettieri G. Incidence of multiple sclerosis in Bagheria City, Sicily, Italy. Neurol Sci. 2000 Dec;21(6):361-5.
16. Rosati G, Aiello I, Mannu L, et al. Incidence ofmultiple sclerosis in the town of Sassari, Sardinia,1965 to 1985: Evidence for increasing occurrence of the disease. *Neurology*1988; 38(3): 384–388.
17. Rosati G, Aiello I, Pirastru MI, Mannu L,Sanna G, Sau GF, Sotgiu S: Epidemiology of multiple sclerosis in Northwestern Sardinia: further evidence for higher frequency in Sardinians compared to other Italians. Neuroepidemiology 1996; 15: 10–19.
18. Sotgiu S, Pugliatti M, Sanna A et al. Multiple sclerosis complexity in selected populations thechallenge of Sardinia, insular Italy. European Journal of Neurology 2002;9: 329–341.
